# *Mycobacterium kansasii* Subtype I Is Associated With Clarithromycin Resistance in China

**DOI:** 10.3389/fmicb.2016.02097

**Published:** 2016-12-26

**Authors:** Yanming Li, Yu Pang, Xunliang Tong, Huiwen Zheng, Yanlin Zhao, Chen Wang

**Affiliations:** ^1^Graduate School, Peking Union Medical CollegeBeijing, China; ^2^Department of Respiratory and Critical Care Medicine, Beijing HospitalBeijing, China; ^3^National Center for Tuberculosis Control and Prevention, Chinese Center for Disease Control and PreventionBeijing, China; ^4^Department of Geriatrics, Beijing HospitalBeijing, China; ^5^Department of Respiratory Medicine, Chinese-Japanese Friendship HospitalBeijing, China

**Keywords:** *Mycobacterium kansasii*, clarithromycin, subtype, drug susceptibility, China

## Abstract

*Mycobacterium kansasii* is the second most common cause of slowly growing non-tuberculous mycobacteria diseases in China. The aim of the present study was to analyze *M. kansasii* subtypes isolated from patients in China, and to explore the antimicrobial susceptibility of the differentiation among these diverse subtypes. A total of 78 *M. kansasii* strains from 16 provinces of China were enrolled in this study. Amikacin (AMK) was the most highly active against *M. kansasii* strains, and only 4 isolates (5.1%) exhibited *in vitro* resistance to AMK. The percentage of levofloxacin (LFX) resistant strains among the 78 *M. kansasii* isolates was 39.7% (31/78), which was significantly higher than that of moxifloxacin (16.7%, *P* = 0.001) and gatifloxacin (19.2%, *P* = 0.005). By using PCR-restriction fragment analysis of the *hsp65* gene (PRA), all the isolates were classified as four different subtypes. Of these four subtypes, *M. kansasii* subtype I was the most frequent genotype in China, accounting for 71.8% (56/78) of *M. kansasii* isolates. Resistance to clarithromycin (CLA) was noted in 26.8% (15/56) of subtype I isolates, which was significant higher than that of other subtypes (4.5%, *P* = 0.031). DNA sequencing revealed that the presence of mutations in 23S rRNA was associated with 56.2% (9/16) of CLA-resistant *M. kansasii* isolates. In conclusion, our data demonstrate that AMK is the most active agent against *M. kansasii in vitro*, while the high proportion of CLA resistance is noted in *M. kansasii* isolates. In addition, the predominant subtype I is associated with CLA resistance in China.

## Introduction

Nontuberculous mycobacteria (NTM) are a large, diverse group of environment organisms that are ubiquitous in the soil, water, animals, human, and food (Griffith et al., 2007; Prevots and Marras, 2015). Despite of low pathogenicity to humans, NTM can cause a wide array of clinical illness, especially in human immune-deficiency virus (HIV) infected patients or those with severely compromised immune systems (Prevots and Marras, 2015). In recent years, increasing attention has been paid to disease caused by NTM because of its more prevalent incidence globally (Hoefsloot et al., 2013; Russell et al., 2014).

Only behind *Mycobacterium avium* complex, *Mycobacterium kansasii* is the second most common cause of NTM diseases in South American and some European countries (Hoefsloot et al., 2013). In addition to the most frequent pulmonary disease, *M. kansasii* is a contributor to various clinical manifestations, including extrapulmonary and disseminated diseases in patients with immunodeficiencies (Campo and Campo, 1997; Pintado et al., 1999). Previous epidemiological studies have demonstrated that patients with chronic lung disease, previous mycobacterial disease, malignancy, and alcoholism are at high risk for *M. kansasii* infection (Guay, 1996). *M. kansasii* is often recovered from tap water, and occasionally from natural water (McSwiggan and Collins, 1974; Steadham, 1980; Taillard et al., 2003). Due to wide exposure to the bacterium, human-to-human transmission of *M. kansasii* is traditionally thought to be uncommon (Ricketts et al., 2014). However, a recent report from Ricketts et al. (2014) observed the potential transmission of *M. kansasii* given the ideal circumstances, providing important insights for public health concern regarding *M. kansasii*.

PCR-restriction fragment analysis of the *hsp65* gene (PRA) is a powerful technique of differentiating heterogeneous subtypes of *M. kansasii* (Telenti et al., 1993; Santin et al., 2004). To date, seven subtypes (I-VII) have been identified based on the PCR-RFLP profiles (Picardeau et al., 1997; Richter et al., 1999; Santin et al., 2004). Of these seven subtypes, subtype I represents the most frequent isolates contributing to human disease (Taillard et al., 2003), and subtype II is likely to be observed in HIV-infected patients, indicating that it may act as an opportunistic agent (Witzig et al., 1995). In contrast, the other five subtypes are generally isolated from environmental samples rather than human specimens (Alcaide et al., 1999). The niche heterogeneity indicates that there may be diverse pathogenicity and biological features among the *M. kansasii* subtypes (Santin et al., 2004). Considering drug susceptibility is an important indicator for predicting the clinical outcome of patients infected with *M. kansasii*, it is meaningful to investigate the assumed difference in drug susceptibility profiles of these seven subtypes. Unfortunately, limited data is known regarding this issue. The aim of the present study was to analyze *M. kansasii* subtypes isolated from patients in China, and to explore the antimicrobial susceptibility of the differentiation among these diverse subtypes.

## Materials and Methods

### Ethics Statement

The protocols applied in this study were approved by the Ethics Committee of Beijing Hospital. The *M. kansasii* isolates obtained from patients were anonymized, processed using strain number only and were not associated with any identifying details.

### *M. kansasii* Isolates

The strains used in this study were collected from national surveillance of drug resistant tuberculosis from 2008 to 2015 (Zhao et al., 2012; Zhang et al., 2015). All the positive mycobacterial cultures were transferred to National Tuberculosis Reference Laboratory for species identification. Conventional species identification was performed with the Löwenstein–Jensen (L–J) medium containing paranitrobenzoic acid (PNB) and thiophen-2-carboxylic acid hydrazide (TCH) (Zhang et al., 2015). The isolates identified as NTM by biochemical method were further divided into species level with sequencing of multiple genes, including 16S rRNA, 65-kD heat shock protein (*hsp65*), RNA polymerase subunit beta (*rpoB*), and 16S-23S rRNA internal transcribed spacer (ITS) sequence (Zhang et al., 2015). Briefly, the crude genomic DNA was extracted from colonies harvested from L–J medium according to previous report. The 50 μl amplification mixtures were prepared as follow: 25 μl 2× GoldStar MasterMix (CWBio, Beijing, China), 0.2 μM of each primer set and 5 μl of genomic DNA. PCR program was performed for 35 cycles (94°C for 60 s, 60°C for 60 s, and 72°C for 60 s). The amplicons were sent to Tsingke Company (Beijing, China) for sequencing service. DNA sequence analysis was performed by alignment with the homologous sequences of the reference mycobacterial strains using multiple sequence alignments^1^. A total of 78 *M. kansasii* isolates confirmed by molecular method were enrolled for further drug susceptibility testing and PRA identification.

### Minimal Inhibitory Concentration (MIC)

Antimicrobial susceptibility testing was performed following the guidelines by the Clinical and Laboratory Standards Institute [CLSI], 2011. Cation-adjusted Mueller-Hinton broth (CAMHB) plus 5% OADC (oleic acid- albumin-dextrose-catalase) was used for broth microdilution testing. A total of twenty antimicrobial agents were selected for MIC determination, including isoniazid (INH), streptomycin (STR), clarithromycin (CLA), amikacin (AMK), rifampicin (RIF), rifabutin (RFB), ethambutol (EMB), linezolid (LZD), moxifloxacin (MOX), levofloxacin (LFX), gatifloxacin (GAT), sulfamethoxazole (SUL), azithromycin (AZM), capreomycin (CAP), tigecycline (TGC), meropenem (MER), minocyline (MIN), cefoxitin (CFX), tobramycin (TOB) and clofazimine (CLO). The concentrations tested ranged from 0.0625 to 128 μg/ml. The breakpoints of antimicrobial agents were recommended by the CLSI:CLA, > 16 μg/ml;RFB, > 1 μg/ml;AMK, > 32 μg/ml;EMB, > 4 μg/ml;LZD, > 16 μg/ml; MOX, > 2 μg/mL;RFB, > 2 μg/ml; and SUL, > 32 μg/ml (Clinical and Laboratory Standards Institute [CLSI], 2011). In addition, the breakpoint of levofloxacin was referenced from a previous report, defined as >2 μg/ml (Hombach et al., 2013). For gatifloxacin, the temporary breakpoint was set as > 2 μg/ml, which was also used as breakpoint values of ciprofloxacin, levofloxacin and moxifloxacin. The reference *M. kansasii* strain (ATCC12478) was enrolled in each batch for quality control purpose.

### PRA Identification

The PRA identification was performed as previously described (Telenti et al., 1993). The 441-bp fragment of the *hsp65* gene was amplified from genomic DNA with primers Tb11 and Tb12. The PCR product was subjected to restriction enzyme analysis with *Bst*E II and *Hae* III. The restriction fragments were identified by electrophoresis on a 3% of agarose gel. The patterns of PRA were evaluated at the SwissPRAsite^2^.

### DNA Sequencing

The region corresponding to domain V of the 23S ribosomal RNA (23S rRNA) gene was amplified with primer F1 (5′-GCAAGGGTGAAGCGGAGAA-3′) and primer R1 (5′-GCACTTACACTCGCCACCTGA-3′). The amplicons were analyzed by DNA sequencing. The DNA sequences were aligned with the homologous sequences of the reference *M. kansasii* strain (ATCC12478) with multiple sequence alignments^3^.

### Statistical Analysis

The differences in proportions of drug resistance between different *M. kansasii* subtypes were evaluated with chi-square test or Fisher’s exact test. Statistical analysis was performed using SPSS, version 15.0 (SPSS Inc., Chicago, IL, USA). The difference was considered to be statistically significant when *P* < 0.05.

## Results

### Drug Susceptibility Testing

**FIGURE 1:**
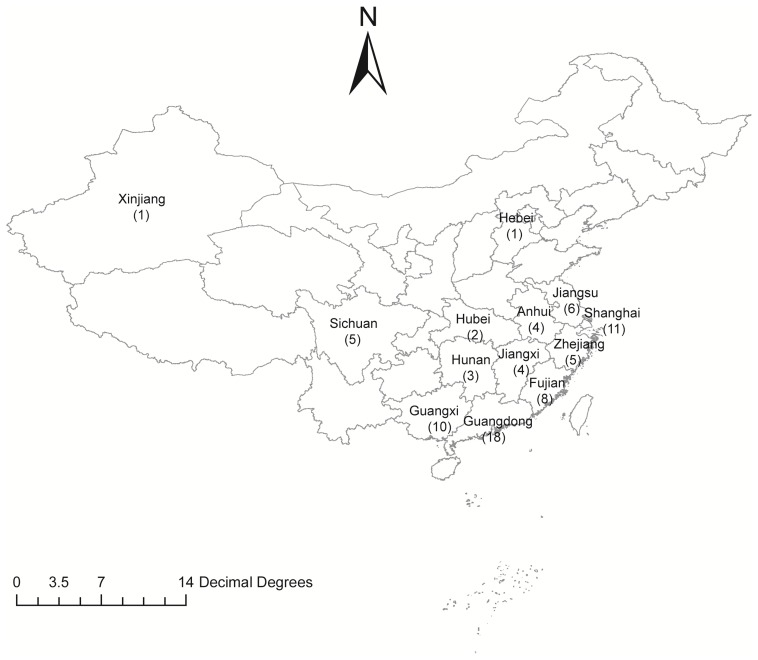
Distribution of *Mycobacterium kansasii* isolates in this study.

**FIGURE 2:**
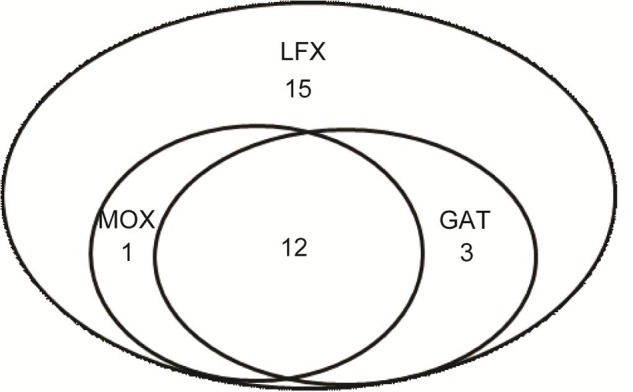
Cross-resistance to fluoroquinolones of *Mycobacterium kansasii* isolates.

A total of 78 *M. kansasii* strains from 13 provinces of China were enrolled for further drug susceptibility testing in this study (**Figure 1**). The range of MICs of each antimicrobial agent for *M. kansasii* isolates is detailed qualitatively in **Table 1**. AMK was the most highly active against *M. kansasii* strains, and only 4 isolates (5.1%) exhibited *in vitro* resistance to AMK. Fluoroquinolone susceptibility was variable among MOX, LFX and GAT, the MIC_50_s and MIC_90_s of which were 0.25 μg/ml and 4 μg/ml for MOX, 2 μg/ml and 16 μg/ml for LFX, and 0.5 μg/ml and 4 μg/ml for GAT, respectively. When using the CLSI breakpoints, the percentage of LFX-resistant strains among the 78 *M. kansasii* isolates was 39.7% (31/78), which was significantly higher than that of MOX (16.7%, *P* = 0.001) and GAT (19.2%, *P* = 0.005). The levels of cross-resistance among fluoroquinolones are presented in **Figure 2**. Of the 31 isolates that were resistant to LFX, 13 (41.9%) and 15 (48.4%) isolates exhibited resistance to MOX and GAT, respectively. In addition, the isolates resistant to MOX and/or GAT were all resistant to LFX. For rifamycins, there were 44 (56.4%) and 27 (34.6%) isolates resistant to RIF and RFB, respectively. Statistical analysis revealed that the percentage of RFB-resistant isolates was significantly lower than that of RIF (*P* = 0.006).

**Table 1:** *In vitro* susceptibility of *Mycobacterium kansasii* isolates against 20 antimicrobial agents enrolled in this study.

Antimicrobial agent	MIC range (μg/ml)	MIC_50_ (μg/ml)	MIC_90_ (μg/ml)	No. of resistant strains (%)^a^
CLA	0.063–128	0.25	128	16 (20.5)
AMK	0.5–64	4	32	4 (5.1)
RIF	0.063–128	2	32	44 (56.4)
RFB	0.063–64	0.25	32	27 (34.6)
EMB	0.13–64	2	32	16 (20.5)
LZD	0.13–128	4	128	25 (32.1)
MOX	0.063–16	0.25	4	13 (16.7)
LFX	0.063–32	2	16	31 (39.7)
GAT	0.063–16	0.5	4	15 (19.2)
SUL	0.25–128	4	128	13 (16.7)
AZM	0.5–128	16	32	–
CAP	0.063–128	1	32	–
TGC	0.25–128	64	128	–
MER	0.25–128	128	128	–
MIN	0.25–128	4	32	–
STR	1–128	8	128	–
CFX	2–128	128	128	–
TOB	0.5–128	8	64	–
CLO	0.06–128	1	64	–
INH	1–64	4	64	–

*^a^INH, isoniazid; STR, streptomycin; CLA, clarithromycin; AMK, amikacin; RIF, rifampicin; RFB, rifabutin; EMB, ethambutol; LZD, linezolid; MOX, moxifloxacin; LFX, levofloxacin; GAT, gatifloxacin; SUL, sulfamethoxazole; AZM, azithromycin; CAP, capreomycin; TGC, tigecycline; MER, meropenem; MIN, minocycline; CFX, cefoxitin; TOB, tobramycin; CLO, clofazimine.*

*^b^MIC50 represents the concentration required to inhibit the growth of 50% of the strains; MIC90 represents the concentration required to inhibit the growth of 90% of the strains.*

We further analyzed the drug susceptibility of *M. kansasii* isolates against antimicrobial agents without the breakpoint values. Out of these ten drugs, CAP showed the most activity against *M. kansasii*, with a MIC_50_ of 1 μg/ml and an MIC_90_ of 32 μg/ml. In contrast, TGC, MER and CFX exhibited poor activity against *M. kansasii*, the MIC_50_s and MIC_90_s of which were higher than 64μg/ml.

### Distribution of Subtypes

All the 78 *M. kansasii* isolates were genotyped by PDA method. As shown in **Table 2**, they were classified as four different subtypes, including subtype I, II, III, and IV. Of these subtypes, subtype I was the most frequent genotype in China, accounting for 71.8% (56/78) of *M. kansasii* isolates. In addition, there were 11 (14.1%), 8 (10.3%), and 3 (3.8%) isolates belonging to subtype II, III, and IV, respectively.

**Table 2:** Distribution of *M. kansasii* classified as different subtypes.

Subtype	No. of isolates (%)	PCR-RFLP profiles of *hsp65*	
		*Bst*E II fragment lengths (bp)	*Hae* III fragment lengths (bp)
I	56 (71.8)	240, 210	140, 105, 80
II	11 (14.1)	240, 135, 85	140, 105
III	8 (10.3)	240, 135, 85	140, 95, 70
IV	3 (3.8)	240, 125, 85	140, 115, 70

### Association between Subtypes and Drug Susceptibility

We further explored the relationship between genetic subtypes and *in vitro* drug susceptibility of *M. kansasii*, which is summarized in **Table 3**. Overall, resistance to CLA was noted in 26.8% (15/56) of subtype I isolates, while only 4.5% (1/22) of isolates from other subtypes was resistant to CLA. Statistical analysis revealed that the proportion of CLA-resistance among subtype I was significantly higher than that among other subtypes (*P* = 0.031). Similarly, subtype I seemed more resistant to EMB (25.0% vs. 9.1%), MOX (33.9% vs. 9.1%), and GAT (23.2% vs. 9.1%) than other subtypes, while the difference was not significant (*P* > 0.05), which might be due to the small sample size. In addition, our data revealed that no statistically significant difference between subtype I and other subtypes in the prevalence of RIF-, RFB-, LZD-, and LFX-resistance (*P* > 0.05).

**Table 3:** Percentage of drug resistance among different subtypes of *M. kansasii* isolates.

Antimicrobial agent	No. of resistant isolates (%)		*P*-value
	Subtype I (56)	Subtype II, III, and IV (22)	
CLA	15 (26.8)	1 (4.5)	0.031
AMK	4 (7.1)	0 (0.0)	0.572
RIF	31 (55.4)	13 (59.1)	0.765
RFB	19 (33.9)	8 (36.4)	0.839
EMB	14 (25.0)	2 (9.1)	0.211
LZD	19 (25.0)	6 (27.3)	0.571
MOX	11 (33.9)	2 (9.1)	0.330
LFX	24 (42.9)	7 (31.8)	0.370
GAT	13 (23.2)	2 (9.1)	0.210
SUL	11 (19.6)	2 (9.1)	0.330

### Mutations in 23S Ribosomal RNA Conferring CLA Resistance

As shown in **Table 4**, the presence of mutations in 23S rRNA was associated with 56.2% (9/16) of CLA-resistant isolates, while 7 (43.8%) of the 16 CLA-resistant isolates were found to have no mutations at positions 2058 and 2059. A total of two types of mutations were observed in 23S rRNA. The most frequent mutation conferring CLA resistance was A-to-T substitution at position 2058, and six isolates harboring this mutation showed resistant to CLA, the MICs of which ranged from 32 to 128 μg/ml. In addition, another substitution at position 2058 (A-to-C) was found in three CLA-resistant isolates.

**Table 4:** Minimal inhibitory concentration (MICs) of CLA and AZM and mutations in the 23S rRNA gene in CLA-resistant *M. kansasii* isolates.

Mutation in 23S rRNA	MIC range of CLA (μg/ml)	MIC range of AZM (μg/ml)	No. of isolates (%)
A2058T	32–128	16–128	6 (37.5)
A2058C	64–128	32–128	3 (18.8)
NA	32–128	4–128	7 (43.8)
Total	32–128	4–128	16 (100.0)

## Discussion

To our best knowledge, the present study is the first provided fundamental information on the drug susceptibility and molecular analysis of *M. kansasii* isolates from China. In keeping with previous reports, our results demonstrated that AMK exhibits favorable *in vitro* activity against *M. kansasii* isolates, which supports its important role in the initial treatment of *M. kansasii* infections (van Ingen et al., 2010; Cowman et al., 2016). Notably, we found that the prevalence of CLA-resistance was 20.5%, which was higher than that from Spain (0%) (Guna et al., 2005), Netherlands (1%) (van Ingen et al., 2010), Brazil (3%) (da Silva Telles et al., 2005), and UK (13%) (Cowman et al., 2016). Due to the satisfactory antibacterial activity and rare adverse effect, macrolides have been used as the first-choice treatment for respiratory tract infection (Yezli and Li, 2012). Numerous literatures have demonstrated that the misuse of macrolides is associated with emergency and increased prevalence of CLA-resistant bacteria in China (Wang et al., 1998, 2001; Chen et al., 2013), which may also serve as a major contributor to the high prevalence of CLA-resistant *M. kansasii* isolates in China. *In vitro* susceptibility of non-tuberculous mycobacteria against CLA exhibits excellent correlation with clinical outcomes (Cowman et al., 2016), the high proportion of CLA-resistant *M. kansasii* in China thus highlights the urgent need to perform *in vitro* CLA susceptibility testing before the initial treatment with regimen containing CLA.

The common *in vitro* resistance to LFX and RIF was observed from our data, indicating the inclusion of these drugs in the therapeutic regimen is controversial. However, the good activity of MOX was detected in this study, which is consistent to previous results from van Ingen et al (van Ingen et al., 2010). A recent study revealed that the combination of MOX and CLA is associated with attenuated CLA activity in a murine model (Kohno et al., 2007). Nevertheless, MOX is an alternative antimicrobial agent for the patients infected with CLA-resistant *M. kansasii*.

Point mutations at either position 2058 or 2059 in the 23S rRNA gene have been reported to confer macrolide resistance phenotypes in NTM species (Inagaki et al., 2011; Zhao et al., 2014). For *M. avium* complex, almost 90% of CLA-resistant isolates harbor nucleotide substitutions in the 23S rRNA gene (Inagaki et al., 2011). In contrast, our data revealed that more than 40% of CLA-resistant *M. kansasii* isolates had no mutation at either position 2058 or 2059 of the 23S rRNA gene. The unsatisfactory correlation between *in vitro* CLA resistance and mutations of 23S rRNA indicates that other mechanisms, such as eﬄux pump and methylation of 23S rRNA, may be involved in CLA resistance for *M. kansasii* (Inagaki et al., 2011; Brown-Elliott et al., 2012).

*Mycobacterium kansasii* subtype I is the most frequent subtype isolated from humans in many geographic areas of the world (da Silva Telles et al., 2005). In agreement with previous reports, the subtype I is also the predominant genotype of *M. kansasii* in China, the prevalence of which is similar to that in Switzerland (68%) (Taillard et al., 2003), although it is lower than that in Spain (98%) (Santin et al., 2004) and Brazil (98%) (da Silva Telles et al., 2005), and higher than that in Germany (33%) (Richter et al., 1999), Poland (40%) (Bakula et al., 2013), and France (43%) (Alcaide et al., 1997). Hence, the distribution of subtype I exhibits geographical diversity, which may be associated with the existence of different ecosystems for these microorganisms (Taillard et al., 2003). In addition, only 14.1% of *M. kansasii* isolates in this study were classified as subtype II, lower than data from previous studies (Alcaide et al., 1997; Richter et al., 1999). Different from Subtype I, Subtype II is considered as an opportunistic agent, which seems to be more frequently isolated from HIV-infected patients (Taillard et al., 2003). Thus, the low prevalence of subtype II may be due to the low epidemic of HIV in China.

Another important finding of the current study is that *M. kansasii* subtype I is associated with CLA resistance in China. Subtype I is the most common cause responsible for human infection by *M. kansasii*, while the other subtypes are likely to be isolated from environmental samples (Taillard et al., 2003). We speculate that the different drug susceptibility profiles between subtype I and other subtypes may reflect their significant difference in ecological niches. Due to more frequent exposure to antibiotic selection pressure from human, subtype I emerges the higher proportion of drug resistance to increase adaptability in hosts. Given that CLA-based drug regimens are effective options for treatment of *M. kansasii* infection, the statistical difference observed in CLA-resistance between subtype I and other subtypes is reasonable. In line to our hypothesis, we also found that subtype I was more likely to be resistant to EMB, MOX, and GAT than other subtypes. Unfortunately, because the small sample size undermines the statistical power, the difference was not statistically significant. Further study with more than 200 isolates will be required to determine the potential relationship between *M. kansasii* subtypes and drug susceptibility profiles.

There were several obvious limitations in this study. First, although chronic pulmonary disease is the most frequent clinical manifestation of NTM infection, it also causes extrapulmonary diseases, such as lymphatic, skin and soft tissue diseases (Griffith et al., 2007). Considering the predominance of *M. kansasii* subtype I in the pulmonary diseases from our observations, it is important to investigate the distribution of difference subtypes in the extrapulmonary infections due to *M. kansasii*. Unfortunately, the isolates enrolled in this study were only collected from suspected pulmonary tuberculosis patients, which impedes the further analysis. Second, HIV status in patients, who are at a high risk for NTM diseases (Henkle and Winthrop, 2015), was not collected. Due to the relative low prevalence of HIV in China, routine HIV examination is not performed among TB patients. On the basis of our findings, future studies will be done to validate the distribution of *M. kansasii* subtypes in these special populations, which will extend our knowledge of host preference and bacterial pathogenicity among various *M. kansasii* subtypes.

## Conclusion

Our data demonstrate that AMK is the most active agent against *M. kansasii in vitro*, while the high proportion of CLA resistance is noted in *M. kansasii* isolates from China. Further PRA analysis reveals that subtype I is the predominant genotype of *M. kansasii* in China. In addition, *M. kansasii* subtype I is associated with CLA resistance. Given the CLA-containing regimen frequently used in the treatment *M. kansasii*, the high proportion of CLA resistance in China highlights the urgent need to perform *in vitro* CLA susceptibility testing before the initial treatment with CLA.

## Author Contributions

YL, YP, YZ, and CW designed this study. YL, YP, and HZ performed experiments. YL, YP, and XT interpreted the data. YL, YP, and CW wrote the manuscript. All authors approved the final version of the paper.

## Conflict of Interest Statement

The authors declare that the research was conducted in the absence of any commercial or financial relationships that could be construed as a potential conflict of interest.

## Footnotes

1 http://www.ncbi.nlm.nih.gov/BLAST

2 http://app.chuv.ch/prasite/index.html

3 http://www.ncbi.nlm.nih.gov/BLAST
